# Cyclic peptide membrane permeability prediction using deep learning model based on molecular attention transformer

**DOI:** 10.3389/fbinf.2025.1566174

**Published:** 2025-03-11

**Authors:** Dawei Jiang, Zixi Chen, Hongli Du

**Affiliations:** ^1^ School of Biology and Biological Engineering, South China University of Technology, Guangzhou, China; ^2^ Department of Gerontology, ShenZhen Longhua District Central Hospital, Shenzhen, China; ^3^ School of Safety Science and Engineering, Anhui University of Science and Technology, Huainan, China

**Keywords:** cyclic peptide, membrane permeability, deep learning, molecular attention transformer, pampa

## Abstract

Membrane permeability is a critical bottleneck in the development of cyclic peptide drugs. Experimental membrane permeability testing is costly, and precise *in silico* prediction tools are scarce. In this study, we developed CPMP (https://github.com/panda1103/CPMP), a cyclic peptide membrane permeability prediction model based on the Molecular Attention Transformer (MAT) frame. The model demonstrated robust predictive performance, achieving determination coefficients (*R* ^*2*^) of 0.67 for PAMPA permeability prediction, and *R* ^*2*^ values of 0.75, 0.62, and 0.73 for Caco-2, RRCK, and MDCK cell permeability predictions, respectively. Its performance outperforms traditional machine learning methods and graph-based neural network models. In ablation experiments, we validated the effectiveness of each component in the MAT architecture. Additionally, we analyzed the impact of data pre-training and cyclic peptide conformation optimization on model performance.

## 1 Introduction

Cyclic peptides have emerged as promising therapeutic candidates owing to their favorable pharmacological properties, including low metabolic toxicity, enhanced stability, high binding affinities, and remarkable efficacy in disrupting protein-protein interactions (Zhang and Chen, 2022; Muttenthaler et al., 2021). Recent advancements in artificial intelligence have significantly enhanced computer-aided design of cyclic peptide drugs, enabling high-throughput screening of cyclic peptides (Kosugi and Ohue, 2023; Rettie et al., 2023). Among the critical factors in cyclic peptide drug development, membrane permeability plays a pivotal role as it directly influences oral bioavailability and intracellular target accessibility (Bockus et al., 2015; Bhardwaj et al., 2022; Hewitt et al., 2015). Traditional experimental approaches for assessing membrane permeability, such as the parallel artificial membrane permeability assay (PAMPA) (Ottaviani et al., 2006), colon epithelial cancer cell (Caco-2) assay (van Breemen and Li, 2005), Ralph Russ canine kidney cell (RRCK) assay (Di et al., 2011), and Madin-Darby canine kidney cell (MDCK) assay (Irvine et al., 1999), are often limited by their time-consuming nature and substantial costs. In response to these challenges, several computational methods have been developed, including MultiCycGT (Cao et al., 2024), PharmPapp (Tan et al., 2024) and CycPeptMP (Li et al., 2024). However, these approaches present notable limitations. MultiCycGT simplifies the prediction of continuous permeability values into a binary classification task, providing only a rough determination of whether cyclic peptides are permeable. The PharmPapp analysis pipeline is specifically designed for the KNIME platform and lacks the flexibility to be extended to mainstream analysis environments. Moreover, its performance is unsatisfactory, with *R* ^*2*^ values ranging from 0.484 to 0.708 for the Caco-2 and RRCK permeability predictions. CycPeptMP, a multi-level molecular feature fusion model, requires specific molecular features as input, which can only be generated using the commercial software MOE.

In this study, we propose the Cyclic Peptide Membrane Permeability prediction model (CPMP), an accessible and open-source solution designed for seamless integration into high-throughput cyclic peptide screening pipelines. Built upon the Molecular Attention Transformer (MAT) neural network (Maziarka et al., 2020), a specialized variant of the Transformer architecture (Vaswani et al., 2017), CPMP leverages interatomic distances and molecular graph structures to enhance its attention mechanism. The MAT framework has previously demonstrated exceptional performance in predicting diverse molecular properties (Maziarka et al., 2020). To predict cyclic peptide permeability, the CPMP model was trained from scratch or fine-tuned using four distinct datasets: PAMPA, Caco-2, RRCK and MDCK. Our results demonstrate that CPMP achieves robust predictive performance, significantly surpassing traditional machine learning methods and other deep learning models across key metrics, including Mean Squared Error (MSE), Mean Absolute Error (MAE), and *R* ^*2*^. This work introduces a powerful computational tool for cyclic peptide membrane permeability prediction, offering substantial potential to accelerate the development of cyclic peptide-based therapeutics.

## 2 Methods

### 2.1 Dataset

The cyclic peptide structures and membrane permeability data were obtained from CycPeptMPDB (Li et al., 2023). Peptide structures were recorded using SMILES notation, and permeability was represented as the log-scaled value, LogP_exp_. Samples with LogP_exp_ < −10.0 were excluded. The permeability data were categorized into four types based on experimental methods: PAMPA, Caco-2, RRCK, and MDCK, with sample counts of 6,701, 1,310, 185, and 64, respectively. For the PAMPA and Caco-2 datasets, we split the data into training, validation, and test sets in a ratio of 8:1:1 (Supplementary Figures S1A, B). For the RRCK and MDCK datasets, the data were divided into training and test sets in a ratio of 7:3 (Supplementary Figures S1C, D). We also analyzed the distribution of six molecular properties, including molecular weight, TPSA (Topological Polar Surface Area), LogP (lipophilicity), ratio of modified amino acids, monomer length in the main chain, and ring count (Supplementary Figures S2–S7).

### 2.2 Architecture

**FIGURE 1:**
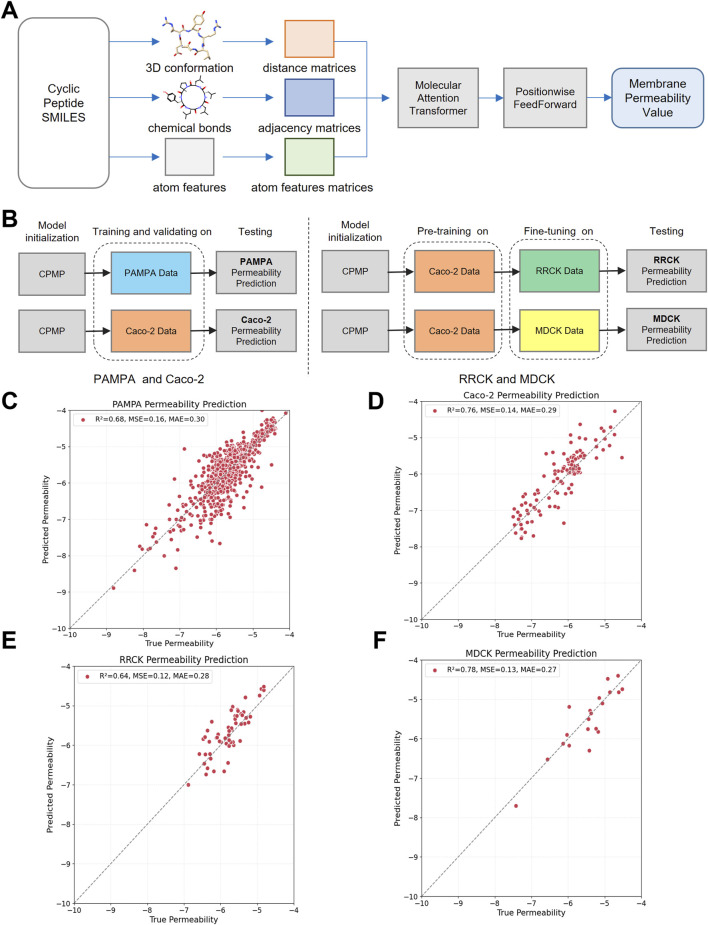
The architecture, training process and testing results of the CPMP model. **(A)** CPMP architecture for predicting cyclic peptide membrane permeability. The CPMP model predicts cyclic peptide membrane permeability from SMILES strings. It processes 3D molecular conformations, bond information, and atom features to construct distance, adjacency, and atom feature matrices. By integrating a molecular attention transformer with position wise feed forward networks, the model generates a permeability value as its output. **(B)** The CPMP model was trained on four different datasets: PAMPA, Caco-2, RRCK, and MDCK. **(C–F)** The testing results of the PAMPA, Caco-2, RRCK, and MDCK membrane permeability prediction model. The number of samples in the testing set are 671, 131, 56, and 20, respectively.

The architecture of the CPMP model is shown in Figure 1A. The core of the CPMP model is MAT (Molecule Attention Transformer) (Maziarka et al., 2020). MAT is a deep learning framework designed for predicting molecular properties. It is based on the Transformer architecture and augmented with molecular graph structure and inter-atomic distances. The framework consists of an embedding layer, multiple Molecule Multi-Head Self-Attention layers, position-wise feed-forward layers, a global pooling layer, and a fully-connected layer for prediction. The attention mechanism in MAT is enhanced by incorporating distance and graph structure information, making it more effective in capturing the complex relationships within molecules. In MAT, the attention scores are computed using three weighted components: the atomic self-attention, distance **(D)**, and adjacency **(A)** matrices, with weights *λ*_*a*_, *λ*_*d*_, and *λ*_*g*_ summing to 1 (Equation 1).

$$A=\left(\lambda_{a}\rho \left(\frac{Q_{i}K_{i}^{T}}{\sqrt{d_{k}}}\right)+\lambda_{d}g\left(D\right)+\lambda_{g}A\right)V_{i},$$ (1)

*g* is the softmax normalization. The optimal *λ* values are determined via grid search. We use MSE (Equation 2) to calculate the loss between the actual (*y*) and predicted (*ŷ*) permeability:

$$Loss=\frac{1}{n}\sum_{i=1}^{n}{\left(y_{i}-{\hat{y}}_{i}\right)}^{2},$$ (2)

### 2.3 Training and testing

The training process is illustrated in Figure 1B. The PAMPA and Caco-2 permeability prediction models are initially trained on the training set, followed by parameter optimization using the validation set, and evaluated on the test set. For the RRCK and MDCK permeability prediction models, the pre-trained Caco-2 model is fine-tuned on the training set, and performance is then evaluated using the test set.

### 2.4 Baseline methods

We validated the performance of CPMP by comparing it with the RFR, SVR and MGNN models. The Random Forest Regressor (RFR) and Support Vector Regression (SVR) models were implemented using scikit-learn (Pedregosa et al., 2011) and utilized 1024-bit Morgan fingerprints generated by RDKit (Landrum et al., 2024) as input features. For the RFR model, the number of trees was set to 100. A series of tree depths, including [5, 10, 15, 20, 25, 30, 35, 40], were tested, and ultimately, 20 was selected as the optimal depth. For the SVR model, we used a Radial Basis Function (RBF) kernel and determined the optimal regularization parameter (C) and tolerance (epsilon) via grid search. Specifically, the search range for C was [0.1, 1, 10, 100], and for epsilon, it was [0.01, 0.1, 0.5, 1]. The model was trained and evaluated using cross-validation for each combination of C and epsilon. The combination that yielded the best performance on the validation set was selected as the optimal set. For the MGNN model, we select its regression task mode. Similarly, the hyperparameters for this model were also determined through grid search, including the dimension of hidden layers in the GNN network, the number of linear layers for fingerprint feature processing, batch size, and learning rate.

### 2.5 Y-randomization test

To assess the risk of chance correlations, we conducted a y-scrambling validation by randomly permuting the cyclic peptides’ permeability labels (Y-values) across the dataset. Following the identical training protocol as our primary models, we retrained 20 scrambled models with distinct label permutations. Performance metrics from these randomized models were then systematically compared with those of the original models to assess whether the observed correlations arose from chance associations.

## 3 Results

### 3.1 Performance of CPMP for PAMPA and Caco-2 permeability prediction

The PAMPA and Caco-2 cell assays are widely used to measure cyclic peptide membrane permeability, providing sufficient data for model training and evaluation (Supplementary Figures S1A, B) (Li et al., 2023). Therefore, we first evaluated the performance of CPMP using PAMPA and Caco-2 data. We compared CPMP with two machine learning models: RFR and SVR (Pedregosa et al., 2011), as well as a deep learning model based on Molecular Graph Neural Networks (MGNN) (Tsubaki et al., 2019).

As shown in Table 1; Figures 1C, D, CPMP outperforms the baseline methods across all evaluation metrics. Specifically, CPMP achieves the lowest MSE of 0.169, significantly better than RFR (0.590), SVR (0.582), and MGNN (0.542). Similarly, the MAE of CPMP (0.308) is lower than those of RFR (0.485), SVR (0.436), and MGNN (0.466), demonstrating its higher accuracy in predicting membrane permeability. Furthermore, CPMP achieves the highest *R* ^*2*^ value of 0.671, indicating stronger explanatory power than RFR (0.388), SVR (0.396), and MGNN (0.546). For Caco-2 permeability prediction, CPMP also shows superior performance. It achieves the lowest MSE of 0.151, better than RFR (0.218), SVR (0.182), and MGNN (0.178). The MAE of CPMP (0.286) is also the lowest among the compared methods, with RFR at 0.349, SVR at 0.322, and MGNN at 0.305. Additionally, CPMP obtains the highest *R* ^*2*^ value of 0.746, indicating a stronger predictive capability compared to RFR (0.643), SVR (0.694), and MGNN (0.702).

**TABLE 1:** Performance comparison between three baseline methods and CPMP for PAMPA and Caco-2 permeability prediction. The metrics are the average values of three repeated runs; the best result for each metric is indicated in bold.

Task	Metrics	RFR	SVR	MGNN	CPMP (MAT)
PAMPA permeability prediction	MSE	0.590 ± 0.003	0.580 ± 0.005	0.542 ± 0.006	**0.169 ± 0.004**
	MAE	0.485 ± 0.001	0.436 ± 0.004	0.466 ± 0.006	**0.308 ± 0.005**
	R^2^	0.388 ± 0.003	0.390 ± 0.005	0.546 ± 0.005	**0.671 ± 0.008**
Caco-2 permeability prediction	MSE	0.218 ± 0.005	0.182 ± 0.006	0.178 ± 0.001	**0.151 ± 0.006**
	MAE	0.349 ± 0.004	0.322 ± 0.010	0.305 ± 0.005	**0.286 ± 0.005**
	R^2^	0.643 ± 0.008	0.694 ± 0.011	0.702 ± 0.001	**0.746 ± 0.010**

**FIGURE 2:**
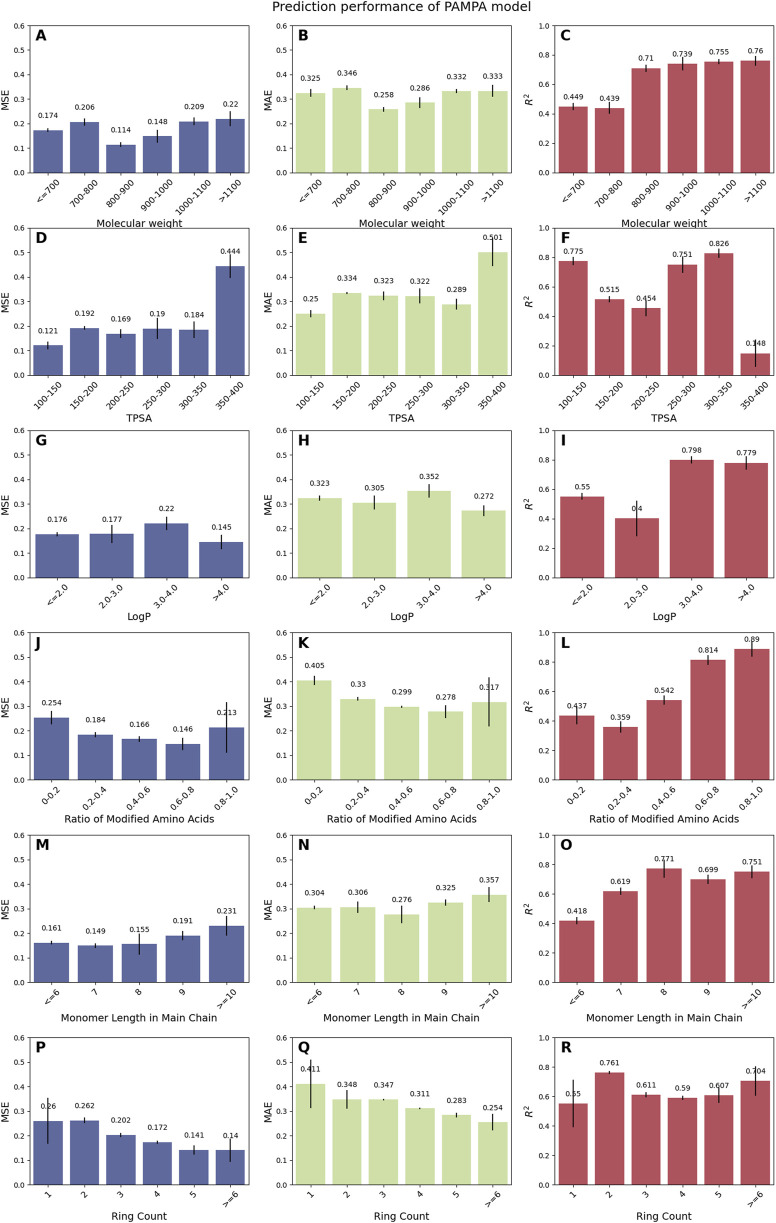
Prediction performance of the PAMPA model across diverse cyclic peptide properties. Peptides were categorized based on molecular weight **(A–C)**, TPSA **(D–F)**, LogP **(G–I)**, the ratio of modified amino acids **(J–L)**, monomer length in main chain **(M–O)**, and ring count **(P–R)**, with each category comprising over 20 samples to ensure robust statistical analysis.

To assess the model’s generalizability, we analyzed its performance across a diverse range of molecular properties, including molecular weight, TPSA, LogP, ratio of modified amino acids, monomer length in the main chain, and ring count. For the PAMPA model (Figure 2), CPMP exhibits robust performance for mid-range molecular weights (800–900 Da: MSE = 0.114, MAE = 0.258, *R* ^2^ = 0.71) and achieves the highest *R* ^2^ (0.76) for larger molecules (>1,100 Da). However, MSE increases slightly for very large (>1,100 Da: MSE = 0.22) or small (≤700 Da: MSE = 0.174) molecules, suggesting room for refinement in extreme size categories. Notably, the model excels for cyclic peptides with moderate TPSA (100–150 Å^2^: *R* ^2^ = 0.775) and high LogP (>4.0: *R* ^2^ = 0.798), while showing reduced accuracy for highly polar molecules (TPSA 350–400 Å^2^: *R* ^2^ = 0.148). For the Caco-2 model (Supplementary Figure S8), CPMP demonstrates strong predictive capability across broad molecular weight ranges (>1,400 Da: MSE = 0.0508, *R* ^2^ = 0.821) and maintains high *R* ^2^ values for peptides with low-to-moderate TPSA (≤300 Å^2^: *R* ^2^ ≥ 0.703). Performance improves significantly for modified amino acid ratios >0.6 (*R* ^2^ = 0.772–0.890), indicating particular strength in modeling heavily modified peptides. While monomer lengths ≥10 show excellent *R* ^2^ (0.784), shorter chains (7–9 units: *R* ^2^ = 0.602) exhibit slightly reduced performance, potentially reflecting imbalanced distribution of training data.

These findings demonstrate that the CPMP model, based on the MAT neural network, performs exceptionally well in delivering accurate and reliable predictions for both PAMPA and Caco-2 permeability. However, there are areas where further improvement is needed, particularly for very large or very small molecules, as well as highly polar molecules. Despite these limitations, the overall performance of CPMP remains superior compared to existing models, highlighting its potential in advancing cyclic peptide drug discovery.

### 3.2 Pre-training enhances performance for RRCK and MDCK permeability prediction

The RRCK and MDCK cell assays are used to study cyclic peptide transmembrane transport in the kidneys. However, the data for RRCK and MDCK are relatively limited (Supplementary Figures S1C, D), which may lead to underfitting during model training. Considering that cyclic peptides from RRCK and MDCK show no significant differences in chemical space compared to those from the Caco-2 dataset (Supplementary Figure S9), we first pre-train the model using Caco-2 data and then fine-tune it with RRCK and MDCK data to enhance its predictive accuracy.

As shown in Table 2; Figures 1E, F, pre-training significantly enhances the predictive capabilities of the CPMP model for RRCK and MDCK permeability predictions. Specifically, for RRCK, the MSE decreases from 0.181 to 0.129, the MAE decreases from 0.328 to 0.288, and the *R* ^*2*^ value increases from 0.470 to 0.623. For MDCK, the MSE decreases from 0.354 to 0.165, the MAE decreases from 0.477 to 0.305, and the *R* ^*2*^ value increases from 0.412 to 0.727.

**TABLE 2:** Comparison of performance between the CPMP model without pre-training and with pre-training for RRCK and MDCK permeability prediction. The metrics are the average values of three repeated runs; the best result for each metric is indicated in bold.

Task	Metrics	CPMP	CPMP with pre-training
RRCK permeability prediction	MSE	0.181 ± 0.004	**0.129 ± 0.005**
	MAE	0.328 ± 0.009	**0.288 ± 0.005**
	R^2^	0.470 ± 0.012	**0.623 ± 0.014**
MDCK permeability prediction	MSE	0.354 ± 0.003	**0.165 ± 0.030**
	MAE	0.477 ± 0.023	**0.305 ± 0.036**
	R^2^	0.412 ± 0.005	**0.727 ± 0.050**

### 3.3 Ablation study and Y-randomization test

In the CPMA model, three key components—the distance matrix, adjacency matrix, and dummy node—are used to characterize molecular features. To assess the impact of each component, we conducted a series of ablation experiments. As shown in Supplementary Table S1, all three components have a significant impact on the model’s performance. In the baseline model, the *R* ^*2*^ values for PAMPA and Caco-2 permeability predictions are 0.671 and 0.746, respectively. Removing the distance matrix results in the largest performance drop, with *R* ^*2*^ decreasing to 0.554 (PAMPA) and 0.556 (Caco-2). MSE and MAE also increase noticeably. Removing the adjacency matrix decreases *R* ^*2*^ to 0.642 (PAMPA) and 0.700 (Caco-2), while MSE and MAE increase slightly. Removing the dummy node decreases *R* ^*2*^ values to 0.628 (PAMPA) and 0.629 (Caco-2), with smaller increases in MSE and MAE. Overall, the distance matrix has the greatest impact, followed by the dummy node, with the adjacency matrix being the least influential but still important.

The Y-randomization test results show that both the PAMPA and Caco-2 models perform significantly better on real data than on scrambled data (Supplementary Figure S10), indicating that the models are reliable and effective. The PAMPA model has an average *R* ^*2*^ value of about 0.67 for real data and 0.10 for scrambled data, while the Caco-2 model has an average *R* ^*2*^ value of about 0.75 for real data and 0.09 for scrambled data.

### 3.4 Comparative analysis of force fields used for conformational optimization

During the passive membrane permeation process, the conformation of cyclic peptides changes from “open” to “close” and then back to “open” (Linker et al., 2023; Noonan et al., 2022). Molecular dynamics simulations indicate that the “close” conformation is the main permeable species (Dougherty et al., 2019). In MAT, distance matrices, which are important input features, are derived from molecular conformations that were optimized using force fields (Maziarka et al., 2020). Ideally, we should find an appropriate molecular force field to optimize and generate the “close” conformation of cyclic peptides. However, the force fields required for molecular dynamics simulations demand high computational resources, making it impractical to simulate nearly ten thousand cyclic peptide molecules. In order to quickly generate the conformation, we tested the two built-in force fields in RDKit (Landrum et al., 2024), the Universal Force Field (UFF) and the Merck Molecular Force Field (MMFF), both with options to consider or ignore non-bonded interactions within the molecule.

As shown in Supplementary Table S1, for PAMPA permeability prediction, UFF-NB achieves the best performance with the highest *R* ^*2*^ value of 0.673. The difference in MSE between the best and worst is approximately 0.006 (UFF-NB vs. UFF + NB), and the difference in MAE is approximately 0.009 (UFF-NB vs. UFF + NB). In contrast, for Caco-2 permeability prediction, MMFF-NB shows the best performance with the highest *R* ^*2*^ value of 0.751. The difference in MSE between the best and worst is approximately 0.004 (MMFF-NB vs. UFF + NB), and the difference in MAE is approximately 0.007 (MMFF-NB vs. UFF-NB). These differences are small relative to the inherent variability in the model’s repeated runs, indicating that the choice of force field parameters has a relatively minor impact on model performance.

## 4 Conclusion

The CPMP model, based on the MAT, shows strong performance in predicting the membrane permeability of cyclic peptides. It achieves high *R* ^*2*^ values of 0.67 for PAMPA, 0.75 for Caco-2, 0.62 for RRCK, and 0.73 for MDCK, outperforming traditional machine learning and other deep learning models. The pre-training on the Caco-2 dataset and fine-tuning on RRCK and MDCK datasets partially alleviates the issue of limited data, improving performance across these datasets and demonstrating the model’s adaptability to different cell lines. Overall, the CPMP model is a promising tool for membrane permeability prediction, aiding in cyclic peptide drug development.

However, the model also has some limitations. Firstly, the PAMPA and Caco-2 models show reduced prediction accuracy for certain molecular properties (Figure 2; Supplementary Figure S9). Both models struggle with larger molecules, as evidenced by higher MSE and MAE values for molecular weights >1,100 in PAMPA and >1,400 in Caco-2. Additionally, the models perform poorly for molecules with high TPSA (>350 for PAMPA and >320 for Caco-2) and extreme LogP values (≤2.0 or >4.0). The PAMPA model also exhibits decreased accuracy for molecules with a high ratio of modified amino acids (>0.8) and longer monomer chains in the main chain (≥10). Secondly, the limited size and imbalance of the RRCK and MDCK datasets may lead to model underfitting, which could undermine its generalizability. Future efforts should focus on data augmentation to enhance model’s generalizability (Chandrasekar et al., 2022; Mone et al., 2023; Dhage et al., 2021). In addition to the challenges mentioned above, the computational resources required for training and predicting with Transformer models also pose a problem. How to utilize parallel computation to address this issue in the future may be an important research direction. Given the increasing demand for efficient and scalable models in various applications, exploring the potential of parallel computation to optimize the training and prediction processes of Transformers could significantly enhance their practicality and broaden their applicability (Zhai et al., 2020; Esfahani et al., 2020; Zhai et al., 2019).

## Data Availability

The original contributions presented in the study are included in the article/Supplementary Material, further inquiries can be directed to the corresponding author.
