# Supplementary material for: Cyclic peptide membrane permeability prediction using deep learning model based on molecular attention transformer
Source: Front Bioinform. 2025 Mar 11;5:1566174. doi: 10.3389/fbinf.2025.1566174 (PMC11933047; doi:10.3389/fbinf.2025.1566174)
Supplement: Supplementary file 1 [file DataSheet1.docx]

Supplementary Information

# Supplementary Figures


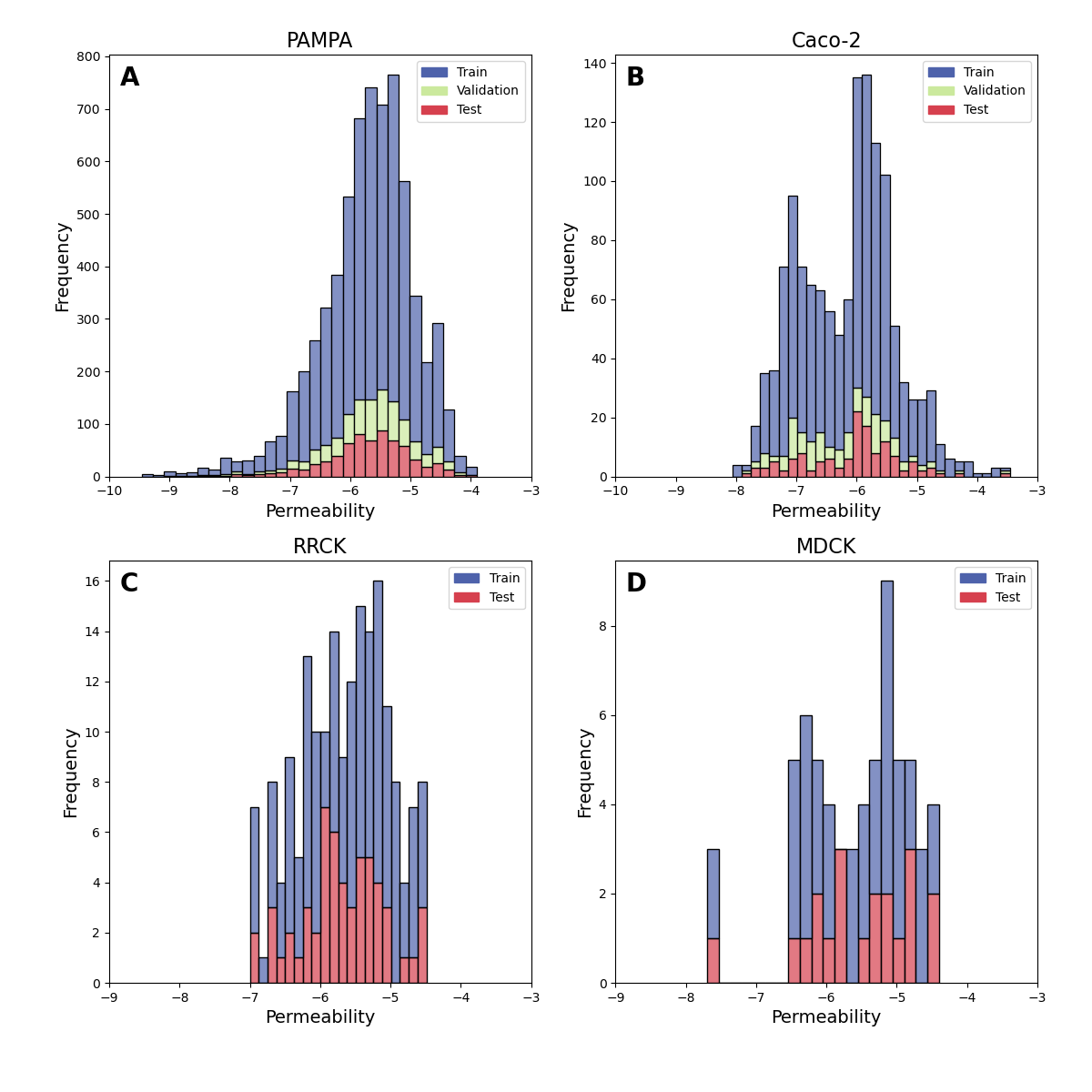


**Figure S1.** Permeability distribution of dataset. **A-B.** For the PAMPA and Caco-2 datasets, we split the data into training, validation, and test sets in a ratio of 8:1:1. **C-D.** For the RRCK and MDCK datasets, the data were divided into training and test sets in a ratio of 7:3.


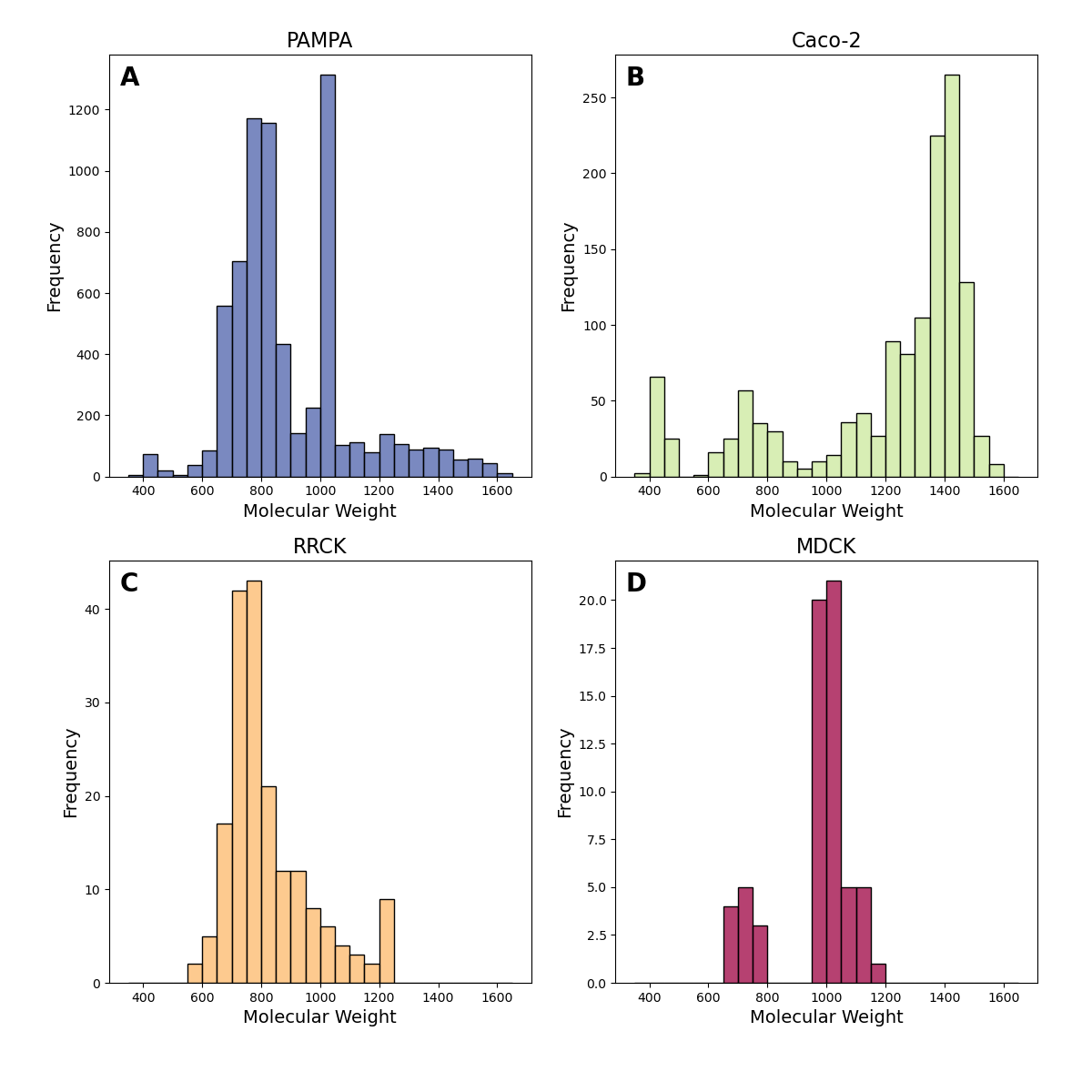


**Figure S2.** Molecular weight distribution of PAMPA, Caco-2, RRCK and MDCK datasets.


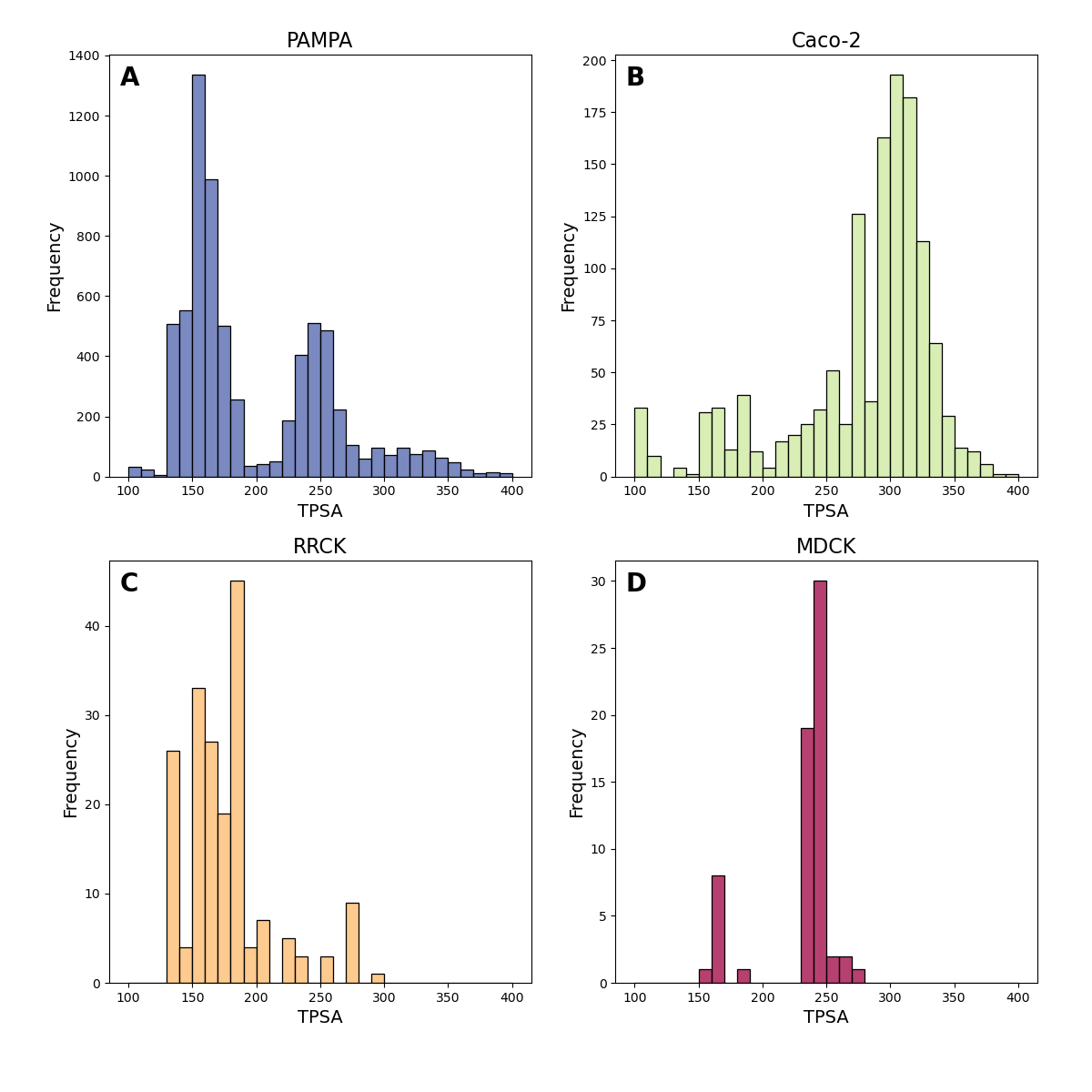


**Figure S3.** Topological Polar Surface Area distribution of PAMPA, Caco-2, RRCK and MDCK datasets


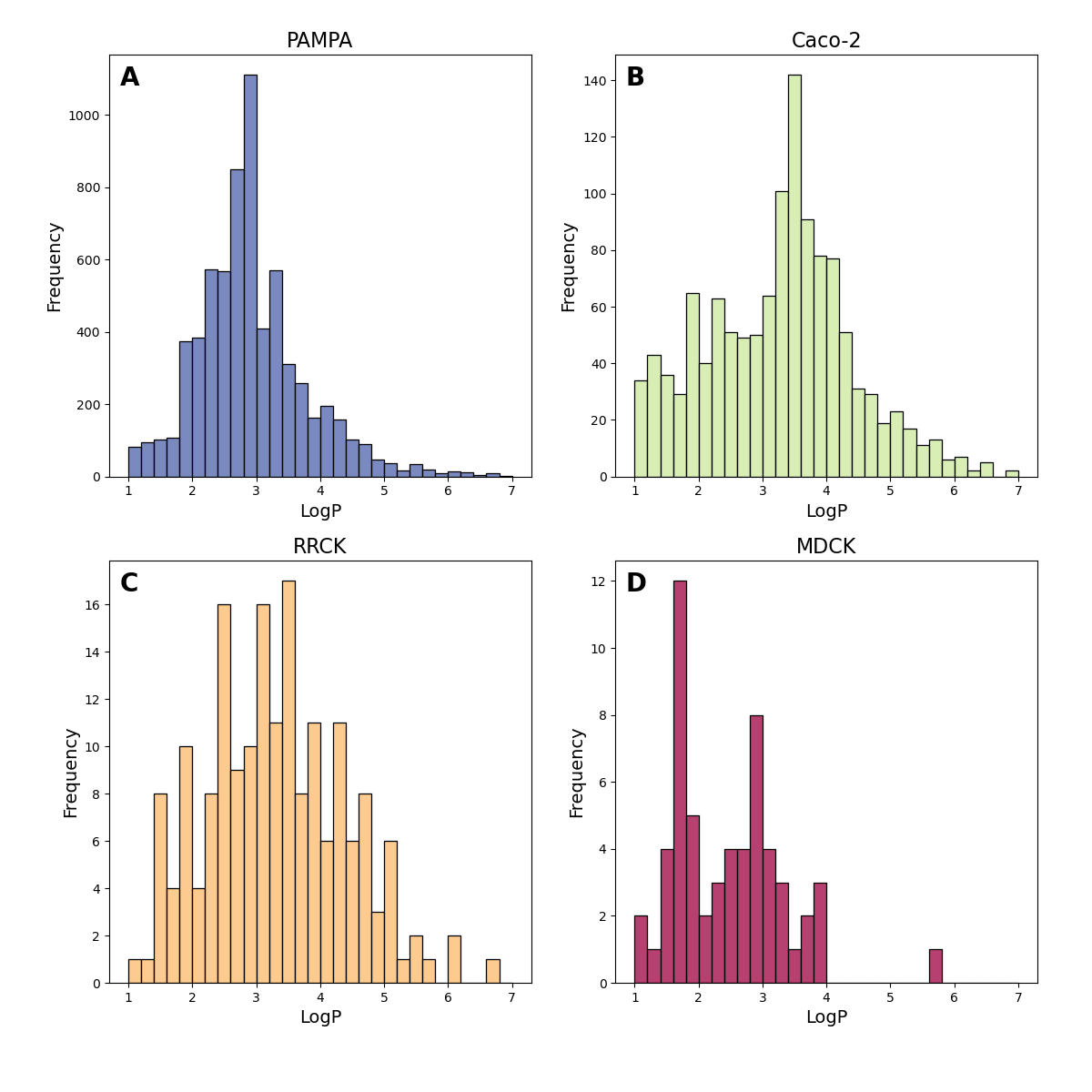


**Figure S4.** LogP distribution of PAMPA, Caco-2, RRCK and MDCK datasets.


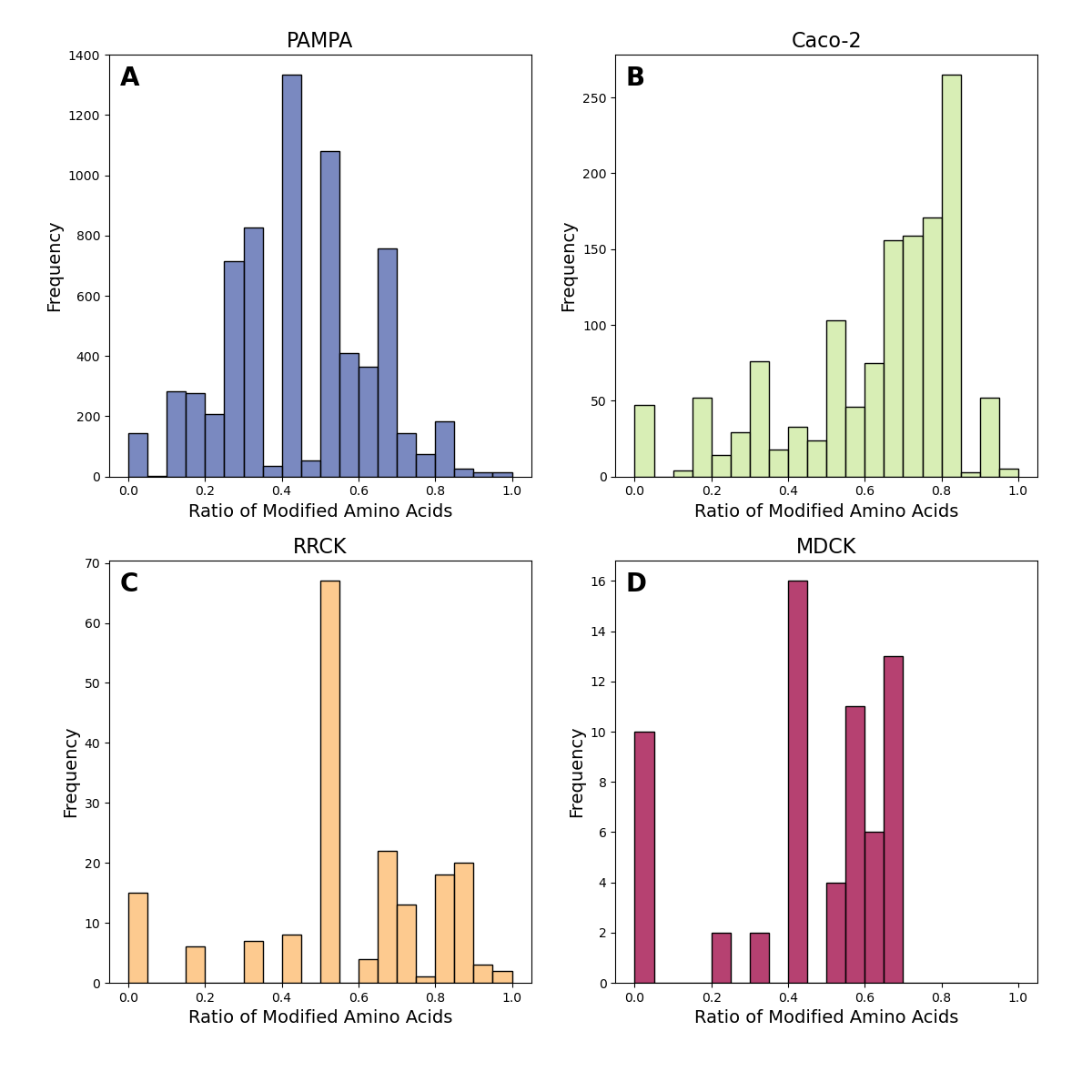


**Figure S5.** The distribution of the ratio of modified amino acids across the PAMPA, Caco-2, RRCK, and MDCK datasets.


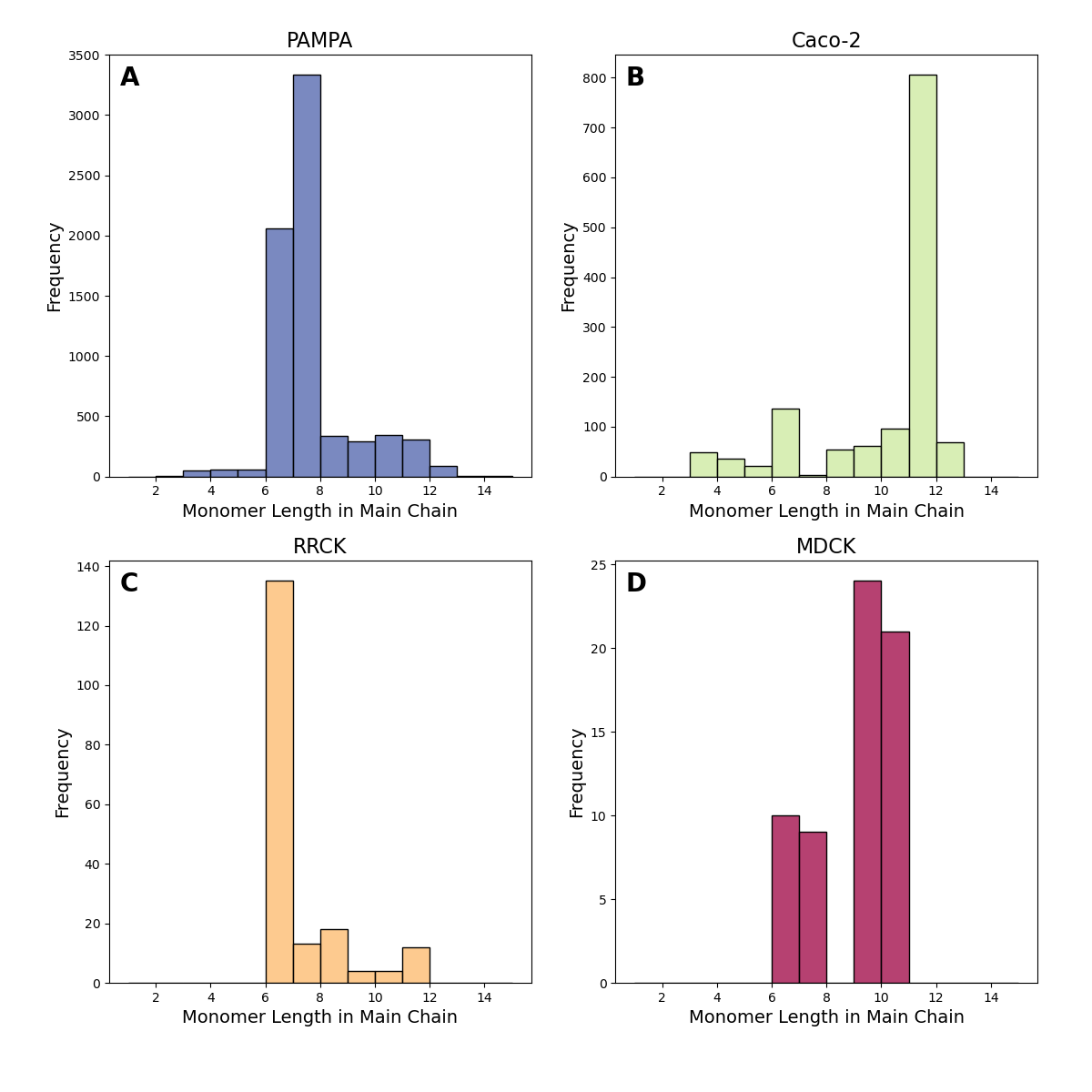


**Figure S6.** The distribution of monomer length in main chain across the PAMPA, Caco-2, RRCK, and MDCK datasets.


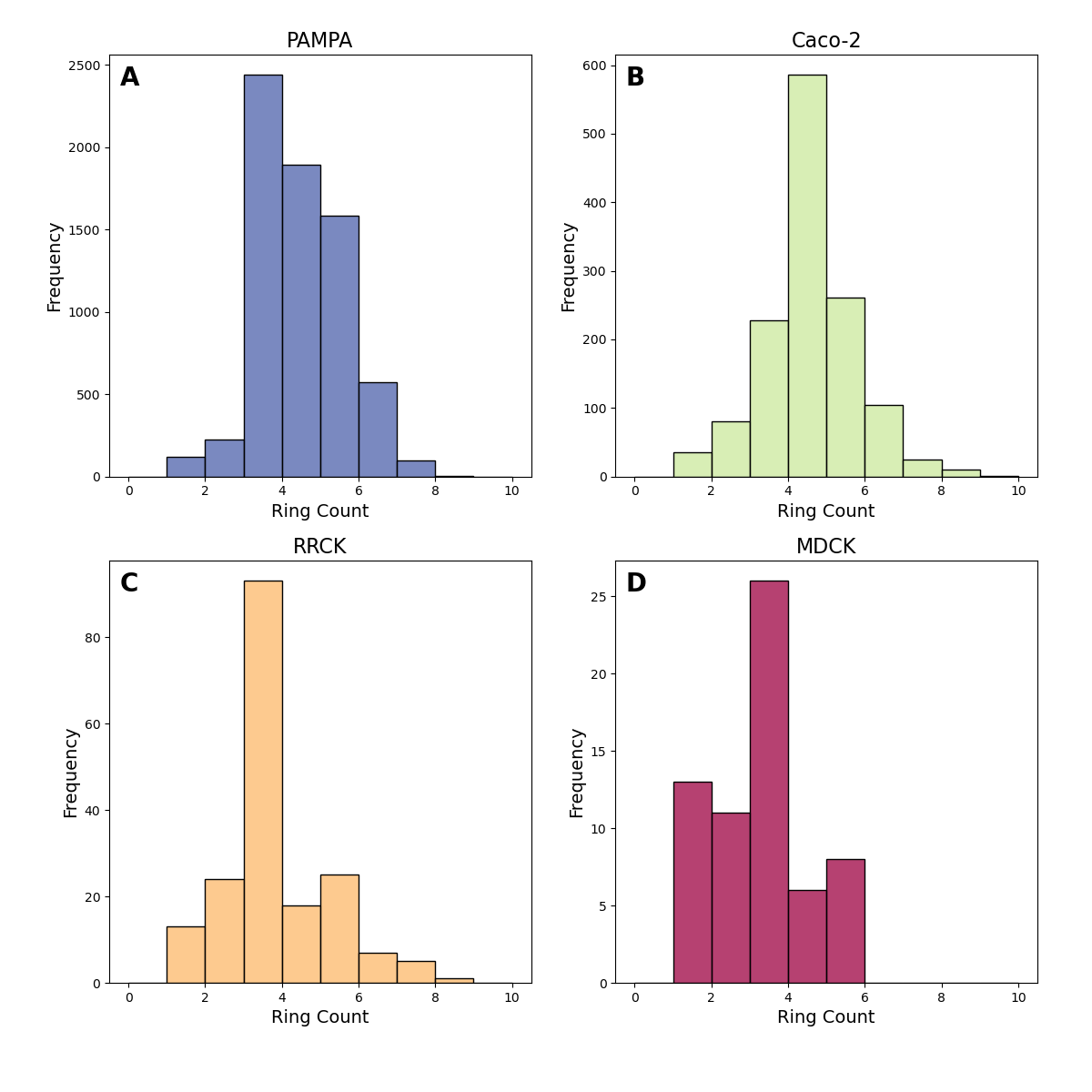


**Figure S7.** Ring count distribution of PAMPA, Caco-2, RRCK, and MDCK datasets.


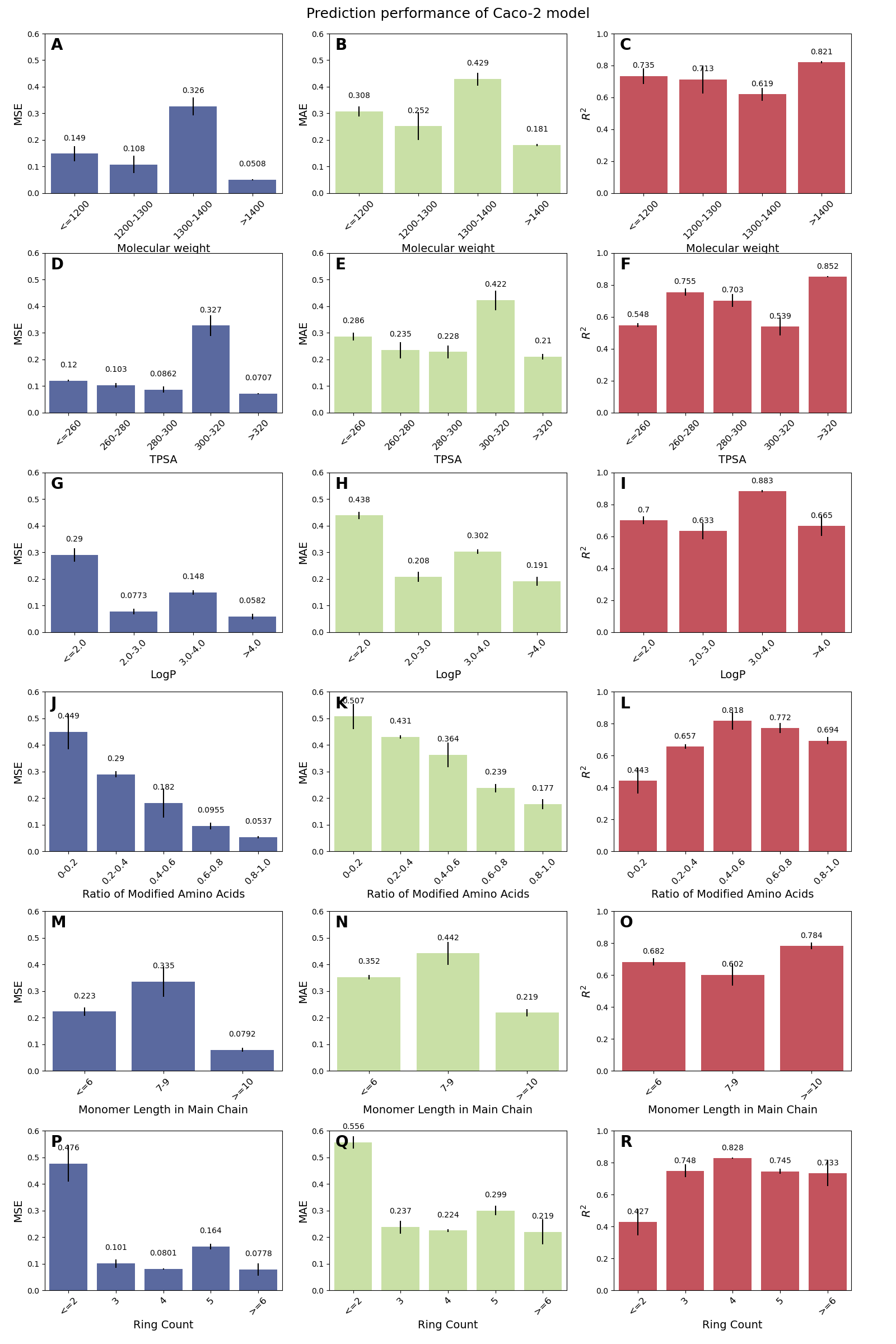


**Figure S8.** Prediction performance of the Caco-2 model across diverse cyclic peptide properties. Peptides were categorized based on molecular weight (**A-C**), TPSA (**D-F**), LogP (**G-I**), the ratio of modified amino acids (**J-L**), monomer length in main chain (**M-O**), and ring count (**P-R**), with each category comprising over 20 samples to ensure robust statistical analysis.


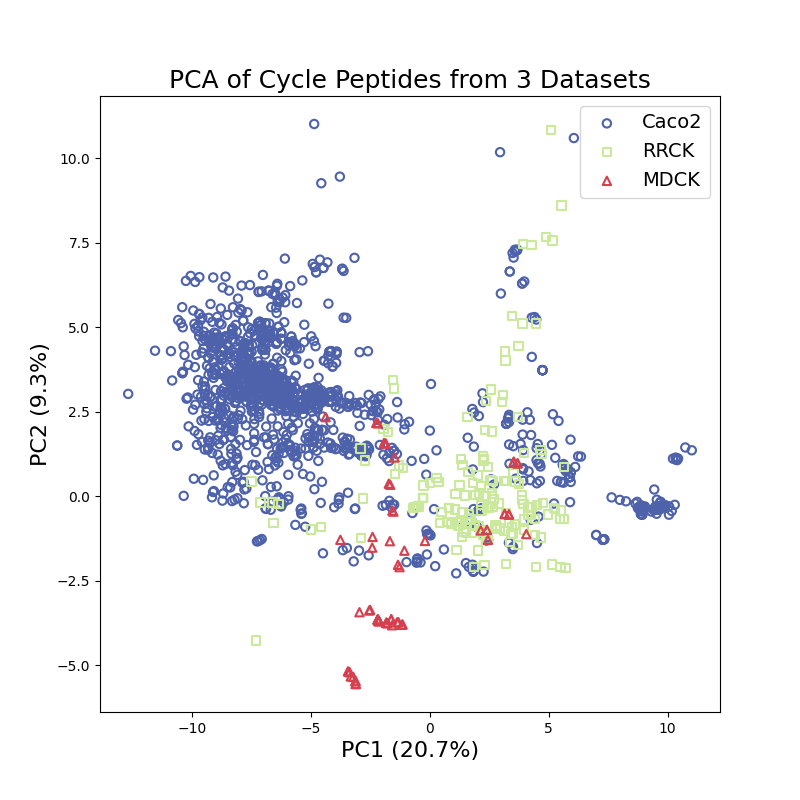
**Figure S9.** The chemical space of cyclic peptides in the Caco-2, RRCK, and MDCK datasets.


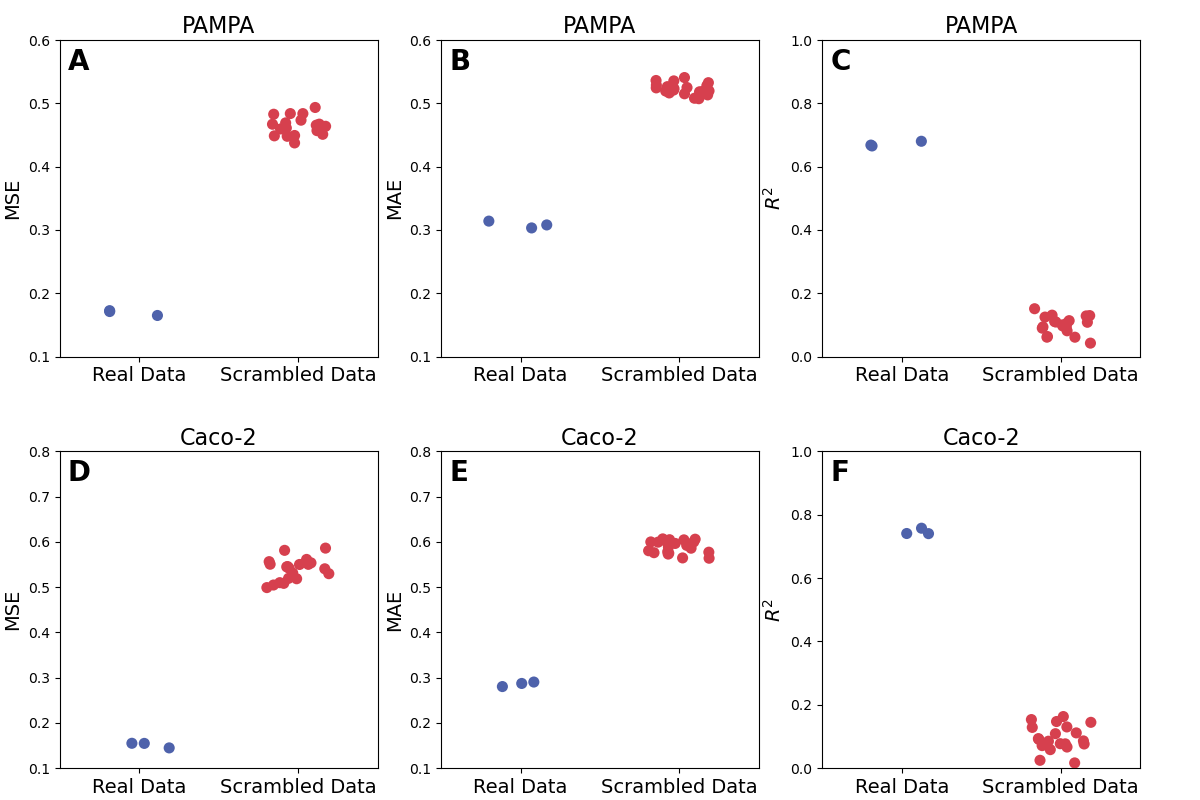


**Figure S10.** Model evaluation with Y-scrambling approach. **A-C** represent the Y-scrambling results of PAMPA models. **D-F** represent the Y-scrambling results of Caco-2 models.

# Supplementary Tables

**Table S1.** Ablation experiments result for the CPMA model. CPMA without distance matrix: set the values of the input distance matrix to 0; CPMA without adjacency matrix: set the values of the input adjacency matrix to 0; CPMA without dummy node: use mean pooling instead of the dummy node method to aggregate different input features. The metrics are the average values of three repeated runs: the best result for each metric is indicated in bold.

| Task | Metrics | CPMP (MAT) | CPMP without distance matrix | CPMP without adjacency matrix | CPMP without dummy node |
| --- | --- | --- | --- | --- | --- |
| PAMPA permeability prediction | MSE | **0.169 ± 0.004** | 0.230 ± 0.004 | 0.184 ± 0.003 | 0.192 ± 0.006 |
|  | MAE | **0.308 ± 0.005** | 0.354 ± 0.002 | 0.317 ± 0.003 | 0.324 ± 0.001 |
|  | R^2^ | **0.671 ± 0.008** | 0.554 ± 0.008 | 0.642 ± 0.006 | 0.628 ± 0.008 |
| Caco-2 permeability prediction | MSE | **0.151 ± 0.006** | 0.265 ± 0.014 | 0.181 ± 0.002 | 0.221 ± 0.017 |
|  | MAE | **0.286 ± 0.005** | 0.397 ± 0.021 | 0.312 ± 0.005 | 0.364 ± 0.016 |
|  | R^2^ | **0.746 ± 0.010** | 0.556 ± 0.024 | 0.700 ± 0.003 | 0.629 ± 0.012 |

**Table S2.** Comparison of performance between the different force field parameters for permeability prediction. UFF: uses RDKit UFF to optimize cyclic peptide’s conformations; MMFF: uses RDKit MMFF to optimize cyclic peptide’s conformations; -NB: ignore non-bonded interactions; +NB: consider non-bonded interactions. The metrics are the average values of three repeated runs; the best result for each metric is indicated in bold.

| Task | Metrics | UFF-NB | UFF+NB | MMFF-NB | MMFF+NB |
| --- | --- | --- | --- | --- | --- |
| PAMPA permeability prediction | MSE | **0.168 ± 0.002** | 0.174 ± 0.005 | 0.171 ± 0.006 | 0.171 ± 0.003 |
|  | MAE | **0.306 ± 0.003** | 0.315 ± 0.005 | 0.309 ± 0.005 | 0.308 ± 0.003 |
|  | R^2^ | **0.673 ± 0.003** | 0.662 ± 0.009 | 0.667 ± 0.012 | 0.668 ± 0.007 |
| Caco-2 permeability prediction | MSE | 0.152 ± 0.007 | 0.153 ± 0.012 | **0.149 ± 0.004** | 0.152 ± 0.005 |
|  | MAE | 0.292 ± 0.006 | 0.287 ± 0.013 | **0.285 ± 0.003** | 0.288 ± 0.005 |
|  | R^2^ | 0.746 ± 0.011 | 0.743 ± 0.020 | **0.751 ± 0.006** | 0.745 ± 0.009 |
